# Supplementary material for: CytoSIP: an annotated structural atlas for interactions involving cytokines or cytokine receptors
Source: Commun Biol. 2024 May 24;7:630. doi: 10.1038/s42003-024-06289-0 (PMC11126726; doi:10.1038/s42003-024-06289-0)
Supplement: Supplementary file 2 — Description of Supplementary Materials [file 42003_2024_6289_MOESM2_ESM.pdf]

Description of Additional Supplementary Files

File name: Supplementary Data 1

Description: symbol, uniprot_id, and full name of Cytokines considered in the current study
