# Supplementary material for: CytoSIP: an annotated structural atlas for interactions involving cytokines or cytokine receptors
Source: Commun Biol. 2024 May 24;7:630. doi: 10.1038/s42003-024-06289-0 (PMC11126726; doi:10.1038/s42003-024-06289-0)
Supplement: Supplementary file 3 — Supplementary Data 1 [file 42003_2024_6289_MOESM3_ESM.pdf]

cytokine

Interleukins

name	uniprot_id	full name
IL3	P08700	Interleukin-3
IL31	Q6EBC2	Interleukin-31
I36RA	Q9UBH0	Interleukin-36 receptor antagonist protein
IL10	P22301	Interleukin-10
IL11	P20809	Interleukin-11
IL12A	P29459	Interleukin-12 subunit alpha
IL12B	P29460	Interleukin-12 subunit beta
IL13	P35225	Interleukin-13
IL15	P40933	Interleukin-15
IL16	Q14005	Pro-interleukin-16
IL17	Q16552	Interleukin-17A
IL17B	Q9UHF5	Interleukin-17B
IL17C	Q9P0M4	Interleukin-17C
IL17D	Q8TAD2	Interleukin-17D
IL17F	Q96PD4	Interleukin-17F
IL18	Q14116	Interleukin-18
IL19	Q9UHD0	Interleukin-19
IL1A	P01583	Interleukin-1 alpha
IL1B	P01584	Interleukin-1 beta
IL1FA	Q8WWZ1	Interleukin-1 family member 10
IL1RA	P18510	Interleukin-1 receptor antagonist protein
IL2	P60568	Interleukin-2
IL20	Q9NYY1	Interleukin-20
IL21	Q9HBE4	Interleukin-21
IL22	Q9GZX6	Interleukin-22
IL23A	Q9NPF7	Interleukin-23 subunit alpha
IL24	Q13007	Interleukin-24
IL25	Q9H293	Interleukin-25
IL26	Q9NPH9	Interleukin-26
IL27A	Q8NEV9	Interleukin-27 subunit alpha
IL27B	Q14213	Interleukin-27 subunit beta
IL32	P24001	Interleukin-32
IL33	O95760	Interleukin-33
IL34	Q6ZMJ4	Interleukin-34
IL36A	Q9UHA7	Interleukin-36 alpha
IL36B	Q9NZH7	Interleukin-36 beta
IL36G	Q9NZH8	Interleukin-36 gamma
IL37	Q9NZH6	Interleukin-37
IL4	P05112	Interleukin-4
IL5	P05113	Interleukin-5
IL6	P05231	Interleukin-6
IL7	P13232	Interleukin-7
IL9	P15248	Interleukin-9

Interferons

name	uniprot_id	full name
IFN10	P01566	Interferon alpha-10
IFN14	P01570	Interferon alpha-14
IFN16	P05015	Interferon alpha-16
IFN17	P01571	Interferon alpha-17
IFN21	P01568	Interferon alpha-21

IFNA1	P01562	Interferon alpha-1/13
IFNA2	P01563	Interferon alpha-2
IFNA4	P05014	Interferon alpha-4
IFNA5	P01569	Interferon alpha-5
IFNA6	P05013	Interferon alpha-6
IFNA7	P01567	Interferon alpha-7
IFNA8	P32881	Interferon alpha-8
IFNB	P01574	Interferon beta
IFNE	Q86WN2	Interferon epsilon
IFNG	P01579	Interferon gamma
IFNK	Q9P0W0	Interferon kappa
IFNL1	Q8IU54	Interferon lambda-1
IFNL2	Q8IZJ0	Interferon lambda-2
IFNL3	Q8IZI9	Interferon lambda-3
IFNW1	P05000	Interferon omega-1

CSF and other factors

name	uniprot_id	full name
CLCF1	Q9UBD9	Cardiotrophin-like cytokine factor 1
CNTF	P26441	Ciliary neurotrophic factor
CSF1	P09603	Macrophage colony-stimulating factor 1
CSF2	P04141	Granulocyte-macrophage colony-stimulating factor
CSF3	P09919	Granulocyte colony-stimulating factor
CSH1	P0DML2	Chorionic somatomammotropin hormone 1
CSH2	P0DML3	Chorionic somatomammotropin hormone 2
CTF1	Q16619	Cardiotrophin-1
EPO	P01588	Erythropoietin
LEP	P41159	Leptin
LIF	P15018	Leukemia inhibitory factor
MIF	P14174	Macrophage migration inhibitory factor
ONCM	P13725	Oncostatin-M
PRL	P01236	Prolactin
SOM2	P01242	Growth hormone variant
SOMA	P01241	Somatotropin
TPO	P40225	Thrombopoietin
TSLP	Q969D9	Thymic stromal lymphopoietin

Chemokines

name	uniprot_id	full name
CC4L	Q8NHW4	C-C motif chemokine 4-like
CCL1	P22362	C-C motif chemokine 1
CCL11	P51671	Eotaxin
CCL13	Q99616	C-C motif chemokine 13
CCL14	Q16627	C-C motif chemokine 14
CCL15	Q16663	C-C motif chemokine 15
CCL16	O15467	C-C motif chemokine 16
CCL17	Q92583	C-C motif chemokine 17
CCL18	P55774	C-C motif chemokine 18
CCL19	Q99731	C-C motif chemokine 19
CCL2	P13500	C-C motif chemokine 2
CCL20	P78556	C-C motif chemokine 20
CCL21	O00585	C-C motif chemokine 21
CCL22	O00626	C-C motif chemokine 22
CCL23	P55773	C-C motif chemokine 23

CCL24	O00175	C-C motif chemokine 24
CCL25	O15444	C-C motif chemokine 25
CCL26	Q9Y258	C-C motif chemokine 26
CCL27	Q9Y4X3	C-C motif chemokine 27
CCL28	Q9NRJ3	C-C motif chemokine 28
CCL3	P10147	C-C motif chemokine 3
CCL4	P13236	C-C motif chemokine 4
CCL5	P13501	C-C motif chemokine 5
CCL7	P80098	C-C motif chemokine 7
CCL8	P80075	C-C motif chemokine 8
CL3L1	P16619	C-C motif chemokine 3-like 1
CXCL2	P19875	C-X-C motif chemokine 2
CXCL3	P19876	C-X-C motif chemokine 3
CXCL5	P42830	C-X-C motif chemokine 5
CXCL6	P80162	C-X-C motif chemokine 6
CXCL7	P02775	Platelet basic protein
CXCL9	Q07325	C-X-C motif chemokine 9
CXL10	P02778	C-X-C motif chemokine 10
CXL11	O14625	C-X-C motif chemokine 11
CXL13	O43927	C-X-C motif chemokine 13
CXL14	O95715	C-X-C motif chemokine 14
CXL16	Q9H2A7	C-X-C motif chemokine 16
CXL17	Q6UXB2	C-X-C motif chemokine 17
CYTL1	Q9NRR1	Cytokine-like protein 1
GP15L	Q6UWK7	Protein GPR15L
GROA	P09341	Growth-regulated alpha protein
IL8	P10145	Interleukin-8
PF4V	P10720	Platelet factor 4 variant
PLF4	P02776	Platelet factor 4
PROK1	P58294	Prokineticin-1
PROK2	Q9HC23	Prokineticin-2
SDF1	P48061	Stromal cell-derived factor 1
X3CL1	P78423	Fractalkine
XCL1	P47992	Lymphotactin
XCL2	Q9UBD3	Cytokine SCM-1 beta

Tumor necrosis fators

name	uniprot_id	full name
CD40L	P29965	CD40 ligand
CD70	P32970	CD70 antigen
EDA	Q92838	Ectodysplasin-A
TN13B	Q9Y275	Tumor necrosis factor ligand superfamily member 13B
TNF10	P50591	Tumor necrosis factor ligand superfamily member 10
TNF11	O14788	Tumor necrosis factor ligand superfamily member 11
TNF12	O43508	Tumor necrosis factor ligand superfamily member 12
TNF13	O75888	Tumor necrosis factor ligand superfamily member 13
TNF14	O43557	Tumor necrosis factor ligand superfamily member 14
TNF15	O95150	Tumor necrosis factor ligand superfamily member 15
TNF18	Q9UNG2	Tumor necrosis factor ligand superfamily member 18
TNFA	P01375	Tumor necrosis factor
TNFB	P01374	Lymphotoxin-alpha
TNFC	Q06643	Lymphotoxin-beta
TNFL4	P23510	Tumor necrosis factor ligand superfamily member 4
TNFL6	P48023	Tumor necrosis factor ligand superfamily member 6

TNFL8	P32971	Tumor necrosis factor ligand superfamily member 8
TNFL9	P41273	Tumor necrosis factor ligand superfamily member 9
TNR14	Q92956	Tumor necrosis factor receptor superfamily member 14

Transforming growth factors (RSTK binding)

name	uniprot_id	full name
BMP1	P13497	Bone morphogenetic protein 1
BMP10	O95393	Bone morphogenetic protein 10
BMP15	O95972	Bone morphogenetic protein 15
BMP2	P12643	Bone morphogenetic protein 2
BMP3	P12645	Bone morphogenetic protein 3
BMP4	P12644	Bone morphogenetic protein 4
BMP5	P22003	Bone morphogenetic protein 5
BMP6	P22004	Bone morphogenetic protein 6
BMP7	P18075	Bone morphogenetic protein 7
BMP8A	Q7Z5Y6	Bone morphogenetic protein 8A
BMP8B	P34820	Bone morphogenetic protein 8B
GDF1	P27539	Embryonic growth/differentiation factor 1
GDF10	P55107	Growth/differentiation factor 10
GDF11	O95390	Growth/differentiation factor 11
GDF15	Q99988	Growth/differentiation factor 15
GDF2	Q9UK05	Growth/differentiation factor 2
GDF3	Q9NR23	Growth/differentiation factor 3
GDF5	P43026	Growth/differentiation factor 5
GDF6	Q6KF10	Growth/differentiation factor 6
GDF7	Q7Z4P5	Growth/differentiation factor 7
GDF8	O14793	Growth/differentiation factor 8
GDF9	O60383	Growth/differentiation factor 9
INHA	P05111	Inhibin alpha chain
INHBA	P08476	Inhibin beta A chain
INHBB	P09529	Inhibin beta B chain
INHBC	P55103	Inhibin beta C chain
INHBE	P58166	Inhibin beta E chain
LFTY1	O75610	Left-right determination factor 1
LFTY2	O00292	Left-right determination factor 2
NODAL	Q96S42	Nodal homolog
TGFB1	P01137	Transforming growth factor beta-1 proprotein
TGFB2	P61812	Transforming growth factor beta-2 proprotein
TGFB3	P10600	Transforming growth factor beta-3 proprotein

Growth factors (RTK binding)

name	uniprot_id	full name
ANGP1	Q15389	Angiopoietin-1
ANGP2	O15123	Angiopoietin-2
ANGP4	Q9Y264	Angiopoietin-4
ARTN	Q5T4W7	Artemin
BDNF	P23560	Brain-derived neurotrophic factor
EFNA1	P20827	Ephrin-A1
EFNA2	O43921	Ephrin-A2
EFNA3	P52797	Ephrin-A3
EFNA4	P52798	Ephrin-A4
EFNA5	P52803	Ephrin-A5
EFNB1	P98172	Ephrin-B1
EFNB2	P52799	Ephrin-B2

EFNB3	Q15768	Ephrin-B3
FGF8	P55075	Fibroblast growth factor 8
FGF9	P31371	Fibroblast growth factor 9
EGF	P01133	Pro-epidermal growth factor
FGF1	P05230	Fibroblast growth factor 1
FGF10	O15520	Fibroblast growth factor 10
FGF11	Q92914	Fibroblast growth factor 11
FGF12	P61328	Fibroblast growth factor 12
FGF13	Q92913	Fibroblast growth factor 13
FGF14	Q92915	Fibroblast growth factor 14
FGF16	O43320	Fibroblast growth factor 16
FGF17	O60258	Fibroblast growth factor 17
FGF18	O76093	Fibroblast growth factor 18
FGF19	O95750	Fibroblast growth factor 19
FGF2	P09038	Fibroblast growth factor 2
FGF20	Q9NP95	Fibroblast growth factor 20
FGF22	Q9HCT0	Fibroblast growth factor 22
FGF23	Q9GZV9	Fibroblast growth factor 23
FGF3	P11487	Fibroblast growth factor 3
FGF4	P08620	Fibroblast growth factor 4
FGF5	P12034	Fibroblast growth factor 5
FGF6	P10767	Fibroblast growth factor 6
FGF7	P21781	Fibroblast growth factor 7
FLT3L	P49771	Fms-related tyrosine kinase 3 ligand
GAS6	Q14393	Growth arrest-specific protein 6
GDNF	P39905	Glial cell line-derived neurotrophic factor
HGF	P14210	Hepatocyte growth factor
IGF1	P05019	Insulin-like growth factor I
IGF2	P01344	Insulin-like growth factor II
NGF	P01138	Beta-nerve growth factor
NRG1	Q02297	Pro-neuregulin-1
NRG2	O14511	Pro-neuregulin-2
NRG3	P56975	Pro-neuregulin-3
NRG4	Q8WWG1	Pro-neuregulin-4
NRTN	Q99748	Neurturin
NTF3	P20783	Neurotrophin-3
NTF4	P34130	Neurotrophin-4
PDGFA	P04085	Platelet-derived growth factor subunit A
PDGFB	P01127	Platelet-derived growth factor subunit B
PDGFC	Q9NRA1	Platelet-derived growth factor C
PDGFD	Q9GZP0	Platelet-derived growth factor D
PLGF	P49763	Placenta growth factor
PSPN	O60542	Persephin
SCF	P21583	Kit ligand
VEGFA	P15692	Vascular endothelial growth factor A
VEGFB	P49765	Vascular endothelial growth factor B
VEGFC	P49767	Vascular endothelial growth factor C
VEGFD	O43915	Vascular endothelial growth factor D
